# Shifts in gut microbiome and metabolome are associated with risk of recurrent atrial fibrillation

**DOI:** 10.1111/jcmm.15959

**Published:** 2020-10-14

**Authors:** Jing Li, Kun Zuo, Jing Zhang, Chaowei Hu, Pan Wang, Jie Jiao, Zheng Liu, Xiandong Yin, Xiaoqing Liu, Kuibao Li, Xinchun Yang

**Affiliations:** ^1^ Heart Center & Beijing Key Laboratory of Hypertension Beijing Chaoyang Hospital Capital Medical University Beijing China; ^2^ Key Laboratory of Upper Airway Dysfunction‐related Cardiovascular Diseases Beijing Institute of Heart, Lung and Blood Vessel Diseases Beijing Anzhen Hospital Capital Medical University Beijing China

**Keywords:** atrial fibrillation, gut microbiota, metabolism, predictive model, recurrence

## Abstract

Alternations of gut microbiota (GM) in atrial fibrillation (AF) with elevated diversity, perturbed composition and function have been described previously. The current work aimed to assess the association of GM composition with AF recurrence (RAF) after ablation based on metagenomic sequencing and metabolomic analyses and to construct a GM‐based predictive model for RAF. Compared with non‐AF controls (50 individuals), GM composition and metabolomic profile were significantly altered between patients with recurrent AF (17 individuals) and non‐RAF group (23 individuals). Notably, discriminative taxa between the non‐RAF and RAF groups, including the families *Nitrosomonadaceae* and *Lentisphaeraceae*, the genera *Marinitoga* and *Rufibacter* and the species *Faecalibacterium* sp*CAG:82*, *Bacillus gobiensis* and *Desulfobacterales bacterium PC51MH44*, were selected to construct a taxonomic scoring system based on LASSO analysis. After incorporating the clinical factors of RAF, taxonomic score retained a significant association with RAF incidence (HR = 2.647, *P* = .041). An elevated AUC (0.954) and positive NRI (1.5601) for predicting RAF compared with traditional clinical scoring (AUC = 0.6918) were obtained. The GM‐based taxonomic scoring system theoretically improves the model performance, and the nomogram and decision curve analysis validated the clinical value of the predicting model. These data provide novel possibility that incorporating the GM factor into future recurrent risk stratification.

## 1 INTRODUCTION

Atrial fibrillation (AF), the commonest arrhythmia among human cardiac diseases, is considered to cause a heavy global burden and directly impair the patient's quality of life.^1^ Medical management of AF with antiarrhythmic medications yields only partial effectiveness and is often associated with multiple adverse effects.^2^ Hence, percutaneous radiofrequency catheter ablation represents an important treatment strategy and option for AF patients, especially individuals showing intolerance or symptomatic disease refractory to antiarrhythmics.^3^ Another issue is therefore raised, as ablation still carries the possibility and risk of AF recurrence (RAF). The success rates of catheter ablation maintaining sinus rhythm and avoiding a recurrence of AF after ablation are hard to predict and control.^4^ To date, multiple variables, such as left atrial diameter and N‐terminal pro‐B‐type natriuretic peptide, are considered risk factors for the recurrence of AF upon catheter ablation; however, these biomarkers lack specificity, and their predictive powers are barely satisfactory.^5^, ^6^ The clinical scoring system, including CAAP‐AF, DR‐FLASH and APPLE scores, could provide a realistic AF ablation outcome expectation for individual patients.^7^, ^8^, ^9^, ^10^, ^11^ However, this scoring system is simple and requires further modifications for increased robustness via substitution of aetiologic factors by surrogate variables. Consequently, developing a novel and better predictive model is quite important.

The alteration and potential function of the gut microbiota (GM) in various pathologies, either as diagnostic biomarkers or as contributors to pathogenesis, have attracted increasing attention.^12^, ^13^ For example, the gut bacterium *Fusobacterium nucleatum* has been identified as a potent biomarker for improving the diagnostic performance of the faecal immunochemical test (FIT), helping detect tumours otherwise missed by FIT. This could allow a step forward in designing a non‐invasive, potentially more accurate and cost‐effective diagnostic tool for advanced colorectal neoplasia.

Recently, we have assessed the role of GM in AF. Our team characterized the associations of GM alterations and metabolic patterns with AF in a previous research.^14^ We further designed a random forest disease classifier based on abundances of co‐abundance gene groups as variables for building a microbiota‐dependent discrimination model for AF detection. However, the correlation between altered GM and AF recurrence after ablation remains unclear. Given the significance of GM shifts in AF patients as previously reported by our team, as well as the risk of AF recurrence following radiofrequency catheter ablation, we wondered whether the GM factor could be applied in predicting the risk of RAF, identifying patients who might benefit more from catheter ablation. Here, we evaluated the profiles of GM and metabolic patterns, assessed AF recurrence after radiofrequency ablation and constructed a GM‐dependent signature to identify the risk of AF recurrence.

## 2 METHODS

### 2.1 Study cohort

Forty non‐valvular AF patients who underwent radiofrequency catheter ablation and 50 non‐AF controls (CTR) were included from our previous study.^14^ Faecal samples were collected before radiofrequency ablation; the gut microbiome was obtained before the ablation procedures were therefore utilized for predicting AF recurrence risk. Metagenomic sequencing results of 50 non‐AF controls' faecal specimens previously assessed by our team were employed as controls. The exclusion criteria for the participants were described in supplementary methods.

### 2.2 Catheter ablation and follow‐up

In general, indications for AF ablation are symptomatic AF refractory or intolerant to one or more Class I or III antiarrhythmics, as well as symptom‐free AF before administration of Class I or III antiarrhythmics.^15^ Upon double‐transseptal puncture under guidance of a 3D‐electroanatomic mapping system, a 3D‐reconstructed image of left atrium was generated with a circular mapping catheter followed by merging to 3D VR cardiac CT scan. Following circumferential pulmonary vein isolation (CPVI), linear ablation and complex fractionated atrial electrogram ablations were appended.^3^ All catheter ablations were carried out by a single surgical team.

The management of patients after ablation was shown in supplementary methods. AF recurrence was defined as any episode of non‐sinus atrial tachyarrhythmia (atrial tachycardia, atrial flutter or AF) lasting more than 30 s and occurring after the three‐month post‐ablation blanking period.^16^ The patients with recurrent AF after ablation would be allocated to the RAF group. And those without recurrence would be classified as the non‐recurrence (non‐RAF) group.

### 2.3 GM assessment by metagenomics

Whole‐metagenome sequencing data of 90 faecal specimens assessed in the current study were obtained from a previous report by our team.^14^ Paired‐end sequencing was carried out on an Illumina Novaseq 6000 (Illumina, USA) with an insert size of 300 bp and a read length of 150 bp. Metagenomic analyses followed the procedures described by our group ^12^, ^13^, ^14^ and shown in the supplementary methods.

### 2.4 GM assessment by metabolomics

Of the above 90 cases, metabolomic data of 60 serum and 52 faecal specimens were available.^14^ Liquid chromatography‐mass spectrometry (LC/MS) was carried out with a Hypercarb C18 column (Thermo Fisher; 3 μm internal diameter, 4.6 × 100 mm) on an UltiMate 3000 chromatography system (Thermo Fisher). Data analysis was carried out as described in our previous reports ^12^, ^14^ and shown in the supplementary methods.

### 2.5 Construction and validation of the predictive model for RAF

The least absolute shrinkage and selection operator (LASSO) method, which has been employed in recent radiomic, genomic, as well as metagenomics studies more than once,^17^, ^18^ was employed for selecting the most efficient predictive indexes from distinctive taxa between the non‐RAF and RAF groups.^19^, ^20^ A taxonomic score (Tax score) was determined for individual patients by linearly combining the retained taxa weighted by the corresponding coefficients. Internal validation followed a reported protocol.^21^ Meanwhile, the mean of 500 bootstrapped estimates of optimism was subtracted from the initial (full cohort model) estimate of the area under curve (AUC) and Nagelkerke R2 to obtain the bootstrap optimism‐corrected estimates of performance.

### 2.6 Ethics statement

The study had approval from the ethics committee of Beijing Chaoyang Hospital and Kailuan General Hospital and the signed informed consent was provided by each participant.

## 3 RESULTS

### 3.1 Characteristics of the study population and follow‐up

In the current study, we included 90 participants from our previous cohort,^14^ with 50 non‐AF CTRs and 40 AF patients. All AF cases underwent radiofrequency catheter ablation before faeces collection, and the patients remained in sinus rhythm (normal beating of the heart) until the end of the procedure, with confirmed CPVI and ablation line blockage.^3^ The occurrence of RAF was regarded as the endpoint. RAF was defined as any episode of non‐sinus atrial tachyarrhythmia, as reported previously.^16^ To date, these AF patients have been followed up for 15.6 ± 12.57 months. RAF was documented in 17 AF patients, with a post‐operative recurrence rate of 42.5%.

The clinical features of patients assessed in this study are summarized in Table 1. Briefly, the baseline clinical characteristics of the non‐RAF and RAF groups were comparable in terms of age, gender, BMI, type 2 diabetes mellitus, hypertension and fasting blood glucose, serum creatinine, total cholesterol and bilirubin amounts. In addition, we determined the CAAP‐AF, DR‐FLASH and APPLE scores, which were significantly higher in the RAF group compared with the non‐RAF group (Table 1; *P* = .043, *P* = .559 and *P* = .564 for the CAAP‐AF, DR‐FLASH and APPLE scores, respectively).

**Table 1:** Baseline clinical characteristics of the study cohort (non‐RAF vs. RAF)

	Non‐RAF	RAF	P value (Non‐RAF vs. RAF)
Number	23	17	/
Age, years	64 (57, 71)	62 (56.5, 71)	0.787
Male/ Female	12/11	28/8	0.107
BMI	26.57 (23.39, 28.44)	26.35 (24.36, 30.25)	0.570
HTN	12	11	0.725
Stroke/TIA/ thromboembolism	3	2	0.957
Carotid artery disease	14	12	0.607
DM	5	5	0.685
TC	4.4 (3.29, 4.80)	3.94 (3.33, 4.56)	0.416
LDL	2.4 (1.5, 3.0)	2.5 (1.6, 2.8)	0.914
FBG	4.65 (4.42, 5.70)	5.26 (4.70, 5.85)	0.165
Creatinine	64.6 (59.6, 77.3)	74.7 (64.05, 86.55)	0.107
TBil	12.1 (9.6, 15.5)	15 (11.15, 21.15)	0.101
LAD	39 (35.75, 44)	42 (39. 47)	0.055
ACEI	3	1	/
ARB	1	2	/
Amiodarone	2	6	/
Propafenone	0	1	/
Statin	1	2	/
DMBG	2	3	/
Oral anticoagulants	6	7	/
CHA2DS2‐VASc score	2 (1, 4)	3 (1.5, 4)	0.766
CAAP‐AF score	3 (2, 5)	5 (4, 7)	0.043
DR‐FLASH score	3 (1, 4)	3 (1, 4)	0.551
APPLE score	2 (1, 3)	2 (0, 2)	0.570

Abbreviations

ACEI, angiotensin‐converting enzyme inhibitors; AF, atrial fibrillation; ARB, angiotensin receptor blockers; BMI, body mass index; CHD, coronary heart disease; DM, diabetes mellitus; DMBG, dimethyl biguanide; FBG, fasting blood glucose; HTN, hypertension; LAD, left atrial diameter; LDL, low‐density lipoprotein; TBil, total bilirubin; TC, total cholesterol; TG, triglyceride; TIA, transient ischaemic attack; UA, uric acid.

CHA2DS2‐VASc score, (congestive heart failure:1; HTN: 1; age > 75:2; T2DM: 1; stroke/TIA/thromboembolism:2; vascular disease: 1; age: 65‐75:1; female: 1). CAAP‐AF score, (coronary artery disease: 1; age: <50:0, 50‐60:1, 60‐70:2, ≥70:3; left atrial size: <4:0, 4‐4.5:1, 4.5‐5:2, 5‐5.5:3, ≥5.5:4; persistent or longstanding AF: 2; antiarrhythmics failed: none:0, 1 or 2:1, >2:2; and female gender: 1). DR‐FLASH score: age ≥ 65 years, persistent AF, impaired estimated glomerular filtration rate (eGFR) (<60 mL/min/1.73 m^2^), left atrium diameter ≥ 43 mm, left ventricular ejection fraction < 50% (1 point for each variable); APPLE score, age ≥ 65 years, persistent AF, impaired eGFR (<60 mL/min/1.73 m^2^), left atrium diameter ≥43 mm, left ventricular ejection fraction <50% (1 point for each variable). IQR, interquartile range; Data are presented as mean ± SD, or median (IQR), as appropriate.

### 3.2 AF recurrence is associated with the dynamically advanced degree of GM dysbiosis

**Figure 1:**
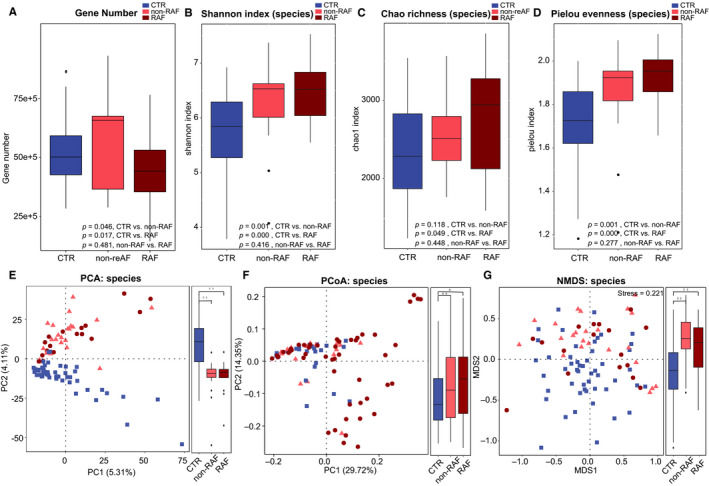
AF recurrence is associated with the dynamically advanced degree of dysbiosis in the GM. Gene number (A) and within individuals (alpha) diversity comprising Shannon index (B), Chao richness (C) and Pielou evenness (D) according to the species profile in non‐AF CTR, non‐RAF and RAF patients. Boxes are interquartile ranges, with lines denoting medians and circles being outliers. Between individuals (beta) diversity comprising PCA (E), PCoA (F) and NMDS (G) according to species abundances. The results depicted a dynamically increasing tendency of diversity among control, non‐RAF and RAF cases. Blue squares represent non‐AF CTR, pink triangles refer to non‐RAF, and red circles denote RAF

The diversity index indicates the variety and richness of microbial entities in the gut and is known to be associated with different disease states.^22^, ^23^, ^24^ To assess gut microbial diversity in RAF patients, total gene amounts (Figure 1A); alpha (within the individual) diversity, comprising Shannon's index (Figure 1B), Chao 1 richness (Figure 1C) and Pielou's evenness (Figure 1D); and beta (between individuals) diversity of principal component analysis (PCA) (Figure 1E), principal co‐ordinate analysis (PCoA) (Figure 1F) and non‐metric dimensional scaling (NMDS) (Figure 1G) plots were analysed at the species level based on metagenomic sequencing data. Compared with the non‐AF CTR group, the non‐RAF and RAF groups had significantly altered alpha and beta GM diversity, except for the Chao 1 index in the non‐RAF group. Although no dynamic discrepancy in microbial diversity was observed between the non‐RAF and RAF groups, there was an increasing and aggravated tendency of shifts in the RAF group, suggesting that patients experiencing recurrence after ablation might possess a more advanced degree of GM dysbiosis than non‐RAF individuals.

### 3.3 Altered gut taxonomic profiles are associated with post‐ablation RAF

The elevated gut microbial diversity and increased degree of GM dysbiosis in RAF indicate the possible overgrowth of some harmful microbes.^25^, ^26^ Thus, the phylogenetic signatures of the GM were analysed with the aim of further examining the changes in GM composition in RAF more specifically (Table S1‐S2). Overall, the non‐RAF and RAF groups shared most microbes detected in the non‐AF CTR group, with 1219 genera (Figure S1A) and 5041 species (Figure S1D). Interestingly, some abundant bacteria, such as the genera *Faecalibacterium* and species *Faecalibacterium prausnitzii*, showed dynamically decreasing tendencies from non‐RAF to RAF. In addition, *Ruminococcus* and *Eubacterium* exhibited progressively increasing trends from the non‐RAF and RAF groups (Figure S1B, C, E, F). These progressive GM shifts associated with recurrent AF confirmed a dynamic and aggravating GM dysbiosis in patients who would suffer from recurrent AF after ablation.

**Figure 2:**
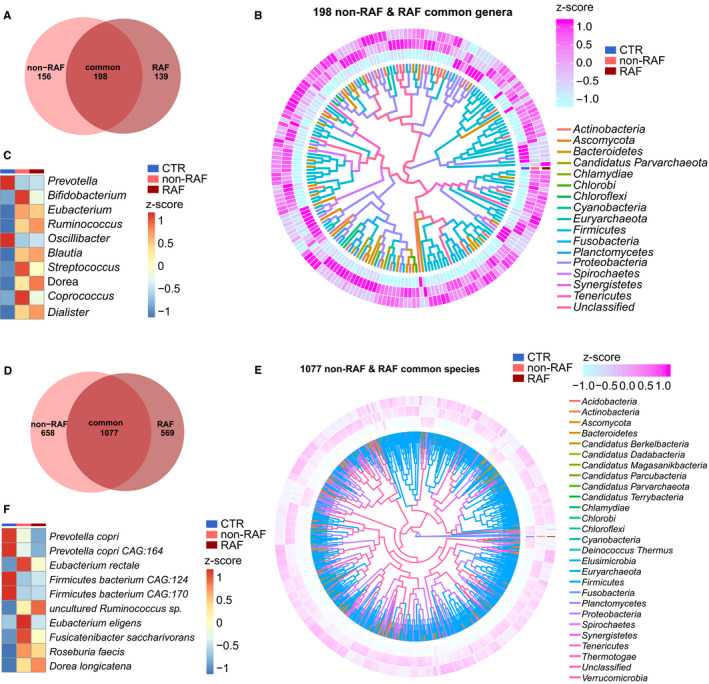
Common taxa in the non‐RAF and RAF groups. A, Venn diagram showing the count of altered genera common to the non‐recurrence of atrial fibrillation (AF) (non‐RAF) (pink) and RAF (red) groups when compared to the non‐AF control (CTR) group. The overlap revealed 198 genera simultaneously detected in AF patients with or without recurrence. B, Heat map revealing 198 commonly altered genera in the non‐RAF and RAF groups when compared to the non‐AF CTR (*q* < 0.05 from Wilcoxon rank‐sum test) and phylogenic associations. Abundance profile is reflected by the z‐score, with genera grouped according to the Bray‐Curtis distance. Negative (blue) and positive (pink) Z‐scores reflect lower and higher abundance levels compared with the mean value, respectively. The colours of the lines inside denote the phyla of given genera. C, Heat map of the first 10 shared genera (*q* < 0.05; Wilcoxon rank‐sum test). Abundance profiles underwent transformation into Z‐scores via average abundance subtraction and division by the standard deviation. Negative (blue) and positive (red) Z‐scores reflected row abundance levels lower and higher compared with the mean, respectively. D, Venn diagram depicting the count of differential species common to the non‐RAF (pink) and RAF (red) groups when compared with the non‐AF CTR group. The overlap revealed 1077 species simultaneously detected in AF patients with or without recurrence. E, Heat map depicting 1077 genera differentially present in the non‐RAF and RAF groups when compared with non‐AF CTR (*q* < 0.05 from Wilcoxon rank‐sum test), and the corresponding phylogenic associations. Abundance profiles were plotted as z‐scores, with genera grouped according to Bray‐Curtis distance. Negative (blue) and positive (pink) Z‐scores reflected row abundance levels lower and higher than the average, respectively. The colours of the lines inside denote the phyla of given genera. F, Heat map of the first 10 shared species (*q* < 0.05; Wilcoxon rank‐sum test). The abundance profiles were analysed as in C. Negative (blue) and positive (red) Z‐scores reflected row abundance levels lower and higher compared with the mean, respectively

Next, we assessed the taxa that were dramatically altered in the gut of non‐RAF volunteers and RAF patients at both the genus and species levels. Compared with the non‐AF CTR group, a total of 354 and 337 genera and 1735 and 1646 species were significantly changed in the non‐RAF and RAF groups, respectively (Table S3). Generally speaking, the non‐RAF and RAF groups shared 198 genera and 1077 species that were simultaneously altered (Figure 2A, D), with most of these common bacteria exhibiting quite a similar tendency in the non‐RAF and RAF groups (Figure 2B, E). Several genera (eg *Prevotella*) and species (eg *Prevotella copri* and *Prevotella copri CAG:164*), which have been documented to be reduced in patients with Parkinson's disease by a previous report,^27^ showed a decreased trend in the non‐RAF group and declined further in the RAF group. Consistently, genera such as *Ruminococcus*, *Blautia*, *Dorea* and *Dialister*, as well as species including *Ruminococcus* sp and *Dorea longicatena*, exhibited a progressively increased trend in patients experiencing recurrence after ablation (Figure 2C, F). *Ruminococcus* exerts pro‐inflammatory effects and contributes to inflammatory bowel disease pathogenesis.^28^ *Ruminococcus* transplantation in germ‐free mice enhances interferon‐γ, interleukin 17 and interleukin 22 amounts.^29^ *Dialister* was shown to be associated with antepartum preeclampsia samples.^30^ Thus, the balanced steady state in the gut is likely broken in AF volunteers, especially in those who suffering from recurrence. The deficiency in health‐promoting bacteria and the enrichment of disease‐causing ones might be associated with RAF pathology after ablation.

Besides the common shifts in gut taxa between the non‐RAF and RAF groups, distinct alterations of bacteria profiles were identified exclusively in non‐RAF or RAF patients. A total of 8 families, 4 genera and 28 species showed significant differences between the non‐RAF and RAF groups (Figure S2). Bacteria such as *Methanobrevibacter* sp, *Methanobrevibacter smithii* and *Methanobrevibacter curvatus* were more abundant in the RAF group, whereas microbes such as *Candidatus* sp, *Phycomycetaceae* sp, *Bacteroidetes bacterium RBG_13_43_22* and *Faecalibacterium* sp *CAG:82* were deficient in the non‐RAF group. We speculated that the shared GM changes in the non‐RAF and RAF groups might represent the core bacterial features of AF, and the unique shifts in GM composition in RAF patients might possibly account for the progression and recurrence of AF.

### 3.4 RAF is associated with disordered metabolomic profiles

The potential mechanisms mediating gut microbial function in human health rely on the interactions of gut microbe‐derived metabolites with target organs.^31^, ^32^ Therefore, metabolomic analyses based on LC/MS were performed to assess the metabolomic profiles of AF patients with or without the risk of AF recurrence following ablation. In this study, samples sufficient for metabonomic analysis were not obtained from all patients. Finally, a subset of 60 participants, comprising 36 non‐AF CTRs, 13 non‐RAF patients and 11 RAF patients, were included in serum metabolite analysis, and 52 individuals (17 non‐AF CTRs, 20 non‐RAF patients and 15 RAF patients) were enrolled for metabolomic profile assessment in the faeces. In serum, 2,500 and 1,733 features in the positive (ESI+) and negative (ESI−) ion modes were obtained, respectively. In faecal samples, 2,549 ESI+ and 1,894 ESI− features were observed. Overall, global metabolic changes in either serum or faeces were revealed between the non‐RAF and RAF groups by both partial least‐squares discriminant analysis (PLS‐DA) and orthogonal PLS‐DA (OPLS‐DA) plots, with significant separation detected between non‐RAF and RAF patients in modes of ES+ and ES− (Figure S3).

**Figure 3:**
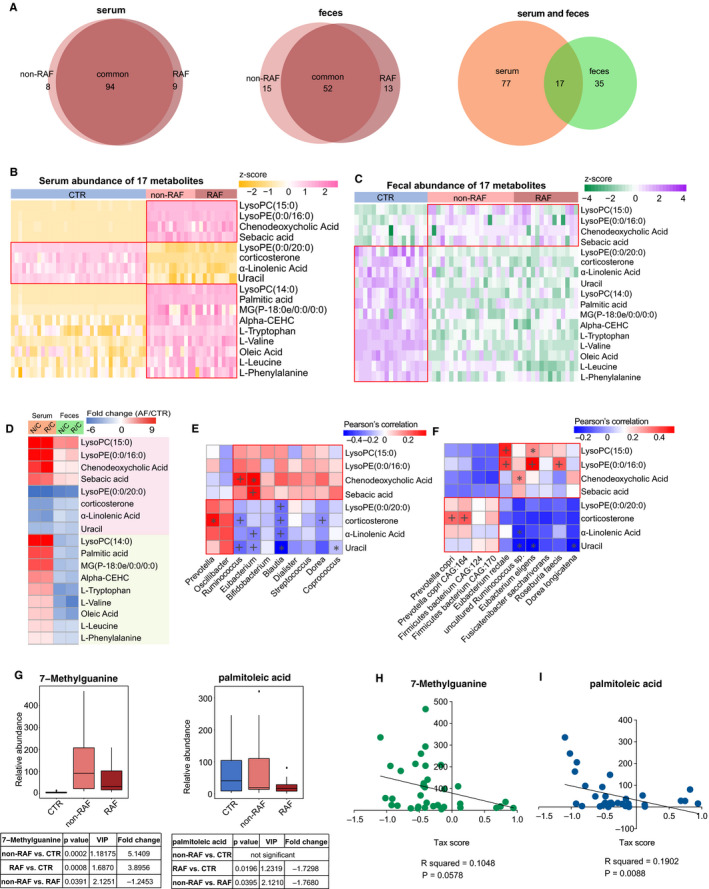
Abnormal metabolic patterns associated with recurrent AF. A, Venn diagram showing the amount of common differential metabolites in the non‐RAF (pink) and RAF (red) groups when compared with the non‐AF control (CTR). The overlap revealed 94 serum and 52 faecal metabolites simultaneously detected in the non‐RAF and RAF groups, whereas 17 endogenous substances were simultaneously found in faecal and serum samples. B and C, Heat map of 17 serum (B) and faecal (C) shared metabolites. Abundance profiles underwent transformation into Z‐scores via average abundance subtraction and division by the standard deviation. Negative (yellow) and positive (pink) Z‐scores reflected row abundance levels lower and higher compared with the mean, respectively. D, Heat map depicting fold changes (AF/CTR) of 17 molecules with alterations in both serum and faecal specimens from AF cases. Fold changes underwent transformation into *t*‐scores. Negative (blue) *t*‐scores reflect compounds showing a decreasing trend in the non‐RAF or RAF groups. Substances increasing or decreasing in both groups (n = 8) or in a single group (n = 9) in faecal and serum specimens are depicted in pink and green, respectively. E and F. Relationship between eight simultaneously altered metabolites and the first 10 commonly detected genera (E) and species (F). As the abundance levels of faecal metabolites mirrored those of GM‐produced substances, faecal metabolomics data underwent Spearman's correlation analysis. Blue, negative correlation; yellow, positive correlation, *P < .05, ^+^ *P* < .01. G, Box plots of two faecal distinctive metabolites between the non‐RAF (pink) and RAF (red) groups. Box, interquartile range; line inside a box, median; circle, outlier. H and I, Correlation between taxonomic (Tax) score and two taxa distinctive between the non‐RAF and RAF groups (*R* ^2^ = .181, *P* = .0023 for 7‐methylguanine; *R* ^2^ = .1217, *P* = .014 for palmitoleic acid. Pearson linear correlations)

Overall, 94 circulating and 52 faecal metabolites showed simultaneous alterations in both non‐RAF and RAF patients compared with non‐AF CTRs (Figure 3A). Interestingly, all 17 metabolites showing overlap in serum and stool specimens (Figure 3B‐D) had similar variation trends in the non‐RAF and RAF groups. Eight of them were synchronously altered in the serum and faeces and thus speculated to constitute the common features and core metabolites associated with AF development, which needs further investigation (Table S4). In addition, two faecal metabolites, 7‐methylguanine and palmitoleic acid, were found to be markedly reduced in cases with RAF in comparison with the non‐RAF group. In addition, 7‐methylguanine and palmitoleic acid showed no higher abundance in the CTR group compared with non‐RAF and RAF cases (Figure 3G).

For assessing the associations of altered metabolites with changed GM, Pearson's correlation analysis was carried out to evaluate the gut genera (Figure 3E) and species (Figure 3F) frequently changed in the non‐RAF and RAF groups, in relation with the eight above‐mentioned representative metabolites. Notably, the metabolites enriched in non‐RAF and RAF patients, including lysophosphatidylethanolamine (LysoPE) (0:0/16:0), chenodeoxycholic acid (CDCA) and sebacic acid, were highly correlated with several AF‐enriched genera (*Ruminococcus* and *Eubacterium*) and species, including *Eubacterium rectale*, *Roseburia inulinivorans* and *Roseburia faecis*. Meanwhile, metabolites deficient in non‐RAF and RAF patients, such as α‐linolenic acid, were negatively correlated with AF‐enriched genera, including *Eubacterium* and *Blautia*. Furthermore, correlation analyses between the two distinctive faecal metabolites and gut microbes showed that non‐RAF enriched 7‐methylguanine and palmitoleic acid were negatively associated with taxonomic (Tax) score decrease in non‐RAF patients (Figure 3H, i. 7‐methylguanine: *R* ^2^ = .1048, 95% CI: −0.593 to 0.01068, *P* = .0578; palmitoleic acid: *R* ^2^ = .1902, 95%CI: −0.6717 to −0.1203, *P* = .0088, respectively). Based on the significant associations of metabolites with gut taxa, the possibility is raised that GM dysbiosis might cause a deficiency in select hear‐protective metabolic products and/or producing deleterious compounds, either directly or indirectly, thereby contributing to the recurrence of AF after ablation. The GM could be, therefore, considered a latent risk factor for RAF.

### 3.5 Development and validation of a predictive model based on GM signature and clinical scores for RAF

**Figure 4:**
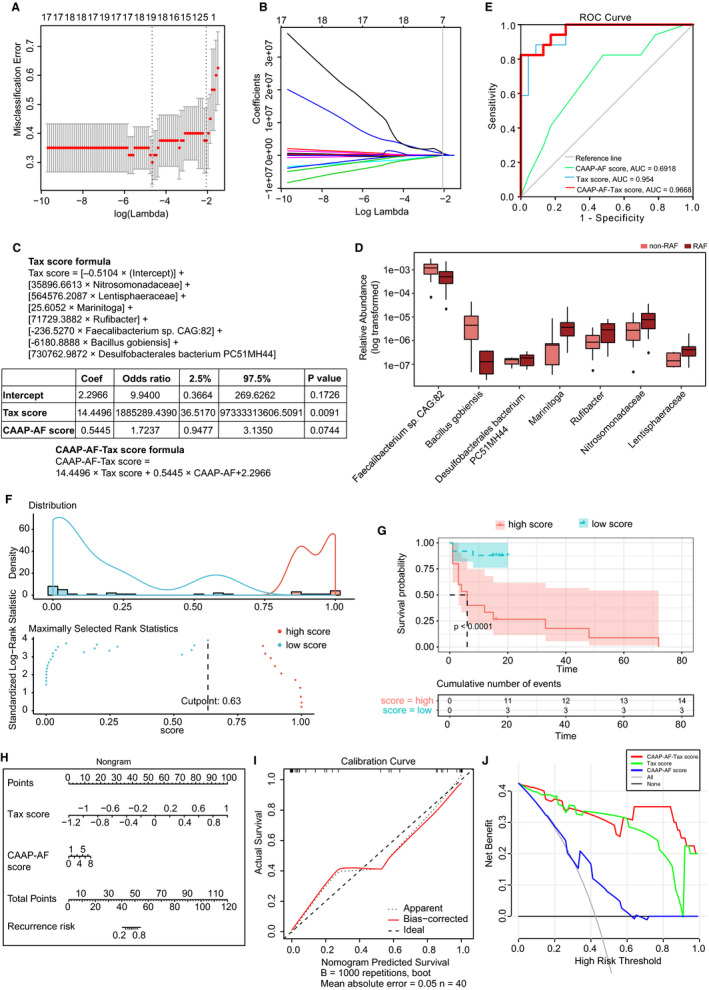
Taxonomic signature to predict recurrence following AF ablation. A, The tuning index (lamda) was selected in the LASSO model receiver operating characteristic curve generation was carried out, and its AUC was plotted against log (lamda). Dotted vertical lines depict the optimal values employing the minimum criteria and 1 standard error of the minimum criteria (1‐SE criteria). A lamda of 0.1268, with log (lamda) of −2.0652 was chosen selected (1‐SE criteria) based on the fivefold cross‐validation method. B, LASSO coefficients of 37 taxonomic features. After excluding highly correlated (|r| ≥ 0.9) taxonomic features and linear combinations, 37 taxonomic features were retained. Coefficients were plotted versus log (lamda). A vertical line is shown at the value determined by fivefold cross‐validation; optimal lamda yielded eight non‐zero coefficients. C, The taxonomic (Tax) score was based on a linear combination of seven taxa‐based markers, and calculated via weighting with their respective coefficients. Logistic regression analysis with the clinical CAAP‐AF score and the developed Tax score was carried out using the enter method. A combined CAAP‐AF‐Tax score formula was constructed by weighting with the respective coefficients. D, Box plots of seven distinctive taxa between the non‐RAF (pink) and RAF (red) groups. Box, interquartile range; line inside a box, median; circle, outlier. E, RAF is identifiable based on the Tax score or CAAP‐AF score. Receiver operating curves for the CAAP‐AF score, Tax score and CAAP‐AF‐Tax score. The areas under the receiver operating curves (AUC values) were as follows: CAAP‐AF score, 0.6918 (95% confidence interval [CI]: 0.525‐0.85, *P* = .04); Tax score, 0.954 (95% CI: 0.8974‐1.000, *P* = .0055); CAAP‐AF‐Tax score, −0.9668 (95% CI: 0.9216‐1.000, *P* = .0011). F, Prognostic information provided by the CAAP‐AF‐Tax score model. Patients were ranked according to increased CAAP‐AF‐Tax score, and maximum difference in overall survival was obtained with a CAAP‐AF‐Tax score = 0.6333, splitting patients into high‐ and low‐risk groups. G, Kaplan‐Meier curves for overall survival prediction by the CAAP‐AF‐Tax score model. Cases were assigned to the high (red)‐ and low (green)‐CAAP‐AF‐Tax score groups according to the corresponding cut‐off CAAP‐AF‐Tax score value of 0.6333. There was a significant difference in overall survival between the high‐ and low‐Tax score groups (*P* < .0001). h. Nomogram for recurrence risk prediction upon catheter ablation based on the Tax score. In the nomogram, each Tax score has a corresponding score on the score scale. A vertical line drawn down the score scale corresponding to the Tax score allows the risk of recurrence in a given patient to be easily and accurately read. I, Calibration curves of the Tax nomogram. Plots show calibrations for various models in terms of agreement between predicted and actual outcome. Model performance is depicted by the apparent plot, and bias correction denotes the corrected value of the deviation, versus the 45‐degree line representing the ideal prediction. J, Decision curve analysis of the Tax score nomogram. The y‐axis reflects the net benefit, with the red line representing the Tax score nomogram; the grey and black lines denote the hypothetical cases with all and no cases exhibiting AF recurrence, respectively. At a threshold probability (patient or doctor)> 1%, employing the Tax score nomogram for AF recurrence prediction shows elevated efficacy compared with the treat‐all‐ or treat‐none schemes. For instance, with an individualized threshold probability of 60% (a patient would be ineligible for therapy with a probability above 60%), a net benefit of 0.3125 is achieved in deciding whether to perform catheter ablation therapy

Subsequently, we sought to establish and assess a predictive model to help make individualized estimates of AF recurrence after ablation. Firstly, we selected the most predictive taxa for RAF by performing LASSO analyses.^19^, ^20^ The results showed that seven bacterial strains consisting of two families (*Nitrosomonadaceae* and *Lentisphaeraceae*), two genera (*Marinitoga* and *Rufibacter*) and three species (*Faecalibacterium* sp*CAG:82*, *Bacillus gobiensis* and *Desulfobacterales bacterium PC51MH44*) among the candidate variables (taxon differing between the non‐RAF and RAF groups) remained statistically significant, with non‐zero coefficients based on 40 AF individuals (Figure 4A, B). Then, we defined a risk score as the Tax score based on a linear combination of these seven taxa‐based markers and calculated the Tax score via weighting with their respective coefficients. The model was constructed as follows: Tax score = [−0.5104 × (Intercept)] + [35896.6613 × *Nitrosomonadaceae*] + [564576.2087 × *Lentisphaeraceae*] + [25.6052 × *Marinitoga*] + [71729.3882 × *Rufibacter*] + [−236.5270 × *Faecalibacterium sp CAG:82*] + [−6180.8888 × *Bacillus gobiensis*] + [730762.9872 × *Desulfobacterales bacterium PC51MH44*] (Figure 4C, D). Patients in the RAF group generally had significantly higher Tax scores (*P* = 3.5973e‐08, Table S5).

In addition, to evaluate whether the GM signature could improve the predictive value of conventional risk factors such as clinical characteristics and drug usage, we performed univariate and multivariate Cox regression analyses, determining hazard ratios (HRs) and respective 95% CIs for parameters showing associations with AF recurrence upon ablation. The diagnostic performance of the model was evaluated by the C index ^33^:0.9‐1.0, outstanding; 0.8‐0.9, excellent; 0.7‐0.8, acceptable.^34^

Because of the limitation of sample size, the CAAP‐AF score was selected as the synthetical reflection of numerous clinical characteristics (including age, gender, left atrial size, AF persistence, antiarrhythmics failed and CAD). We found that the Tax score and statin usage were significantly associated with RAF (Tax score, HR = 2.5, 95% CI: 1.1‐5.8, *P* = .026; Statin usage, HR = 4.8, 95% CI: 1.3‐17, *P* = .019). Meanwhile, other medication factors, including ACEI, ARB, CCB, β‐blocker, propafenone and amiodarone administration, were not significantly associated with RAF (Table S6).

We next carried out multivariate‐adjusted Cox regression based on the above‐mentioned indexes to assess whether GM could improve approaching utilizing conventional clinical factors. Thus, a clinical model incorporating the CAAP‐AF score and statin usage, as well as a combined model including the CAAP‐AF score, statin usage and Tax score were built (Table S6). After incorporating the clinical factors of RAF, Tax score retained a significant association with RAF incidence (HR = 2.647, 95% CI: 1.038‐6.749, *P* = .041). Notably, the combined model had excellent (C index = 0.8329, 95% CI: 0.7249‐0.9410) and significant (*P* = .0428) improvement in performance, in comparison with the clinical model (C index = 0.7261, 95% CI: 0.5813‐0.8709).

Then, using the enter method, logistic regression analysis with the clinical CAAP‐AF score and the developed Tax score was carried out. The Tax score was identified as an independent predictor (Tax score: Coef = 14.4496, odds ratio [OR]=1 885 289.4390, 95% CI: 36.5170‐9.73 + 10; *P* = .0091; CAAP‐AF score, Coef = 0.5445, OR = 1.7237; 95% CI: 0.9477‐3.1350; *P* = .0744) (Figure 4 c). Then, a combined predictive model containing two predictive scores, named CAAP‐AF‐Tax score, was constructed as follows: CAAP‐AF‐Tax score = 14.4496 × Tax score + 0.5445 × CAAP‐AF + 2.2966, with values ranging from −12.4340 to 18.0986 (Table S5). Patients with a score of −12.4340 and 18.0986 had predicted recurrence risks of 3.98e‐04% and 1.00e + 2%, respectively (Figure 4C).

To assess the predictive value of the CAAP‐AF‐Tax model, the AUC based on the ROC curve was determined and compared with those of the CAAP‐AF and Tax scores. Notably, compared with the AUC for the CAAP‐AF score alone (AUC = 0.6918, 95% CI: 0.525‐0.85, *P* = .04), AUC for either the Tax score model (AUC = 0.954, 95% CI: 0.8974‐1.000, *P* = .0055) or CAAP‐AF‐Tax model (AUC = 0.9668, 95% CI: 0.9216‐1.000, *P* = .0011) was significantly higher (Figure 4E). The predictive model was subsequently validated using the net reclassification index (NRI). The NRI after adding the CAAP‐AF score to the Tax score was 1.1509 (*P* = .0003), whereas that after adding the Tax score to the CAAP‐AF score was 1.5601 (*P* = 1.0735e‐06). Therefore, the Tax score theoretically improved the CAAP‐AF model for performance.

Then, the Kaplan–Meier method and log‐rank test were carried out for assessing the prognostic capacities of the developed CAAP‐AF‐Tax model, and AF cases were assigned to high‐ and low‐CAAP‐AF‐Tax score groups according to the optimal cut‐off of 0.633286. There was a significant difference in overall survival between the high‐ and low‐score groups (*P* < .0001, Figure 4F, G).

To provide a quantitative method for predicting individual risk and RAF probability, a nomogram of the CAAP‐AF‐Tax model was established (Figure 4H) and submitted to internal validation as previously reported.^21^ The results showed C index = 0.9668 (95% CI: 0.9155‐1). The calibration curve in Figure 4I demonstrated good agreement with the probability of RAF. The Hosmer‐Lemeshow test revealed non‐statistical significance (*P* = .6318) and suggested inconsiderable departure from the perfect fit.^35^ Subsequently, in Figure 4J, to determine the clinical value of the above Tax nomogram, we carried out decision curve analysis (DCA) via net benefit quantitation at various threshold probabilities.^36^ We found that at a threshold probability (patient or doctor) of 1%, Tax score or CAAP‐AF‐Tax score nomogram use to predict RAF would provide more benefits compared with the treat‐all‐ or treat‐none schemes. Thus, the developed and validated predictive model might be a reliable method for RAF prediction, and help clinicians identify candidates who may benefit from future ablation therapy.

## 4 DISCUSSION

In the current study, we have acquired a series of intriguing results describing the profiles of altered GM and metabolic patterns in AF patients more likely to experience recurrence following radiofrequency ablation. We identified a gradually increasing degree of gut dysbiosis from non‐AF to RAF. Meanwhile, imbalanced metabolic alterations were observed, which indicates the possible GM function in eliciting AF recurrence via interactions with metabolites. This feature of GM is therefore suggested to be a potent risk biomarker for the selection of patients who would benefit from radiofrequency catheter ablation. It is recommended to include an additional focus on GM profiling in the future development of ablation risk stratification and strategies.

Strikingly, this study demonstrated that disordered GM constituted an independent risk factor for AF recurrence. Catheter ablation is an efficient therapeutic option for AF and has been widely used in clinical settings. However, a high post‐ablation recurrence rate calls for identifying novel markers that would enable an improved selection of patients who could benefit from ablation. PV reconnection and unrecognized or progressive extra‐PV AF substrate constitute the immediate reflection of recurrence, but is neither necessary nor sufficient for RAF occurrence.^37^ Therefore, based on the electro‐physiological substrate behind AF persistence and progression, it remains difficult to identify beyond clinical AF parameters.

In our previous research, disordered GM was shown to be associated with the development of AF.^14^ Interestingly, the present study indicated an incremental prognostic accuracy over clinical predictors of the novel predictive model based on GM taxonomic profiles. The newly defined Tax score based on taxonomic profiles in the current work independently predicted AF recurrence, and findings from nomogram and decision curve analyses further confirmed its clinical value. Therefore, GM should be considered a potent predictive model for selecting patients for ablation, and additional focus on disordered GM profiles is strongly recommended in future ablation risk stratification. Although the number of samples included in the current research was small—and hence the robustness of the novel predictive model may not be strong enough—it provides preliminary results and offers a novel concept. Further validation studies with increased sample sizes could improve the overall universality.

Besides the distinctive taxa contained in the predictive score, two distinctive metabolites identified between non‐RAF and RAF were significantly correlated with the Tax score. Although studies describing the direct protective effects of 7‐methylguanine and palmitoleic acid against AF are scarce, investigators have identified the potential roles of 7‐methylguanine and palmitoleic acid in the pathophysiological process related to AF. 7‐methylguanine, a nucleotide contributing to the metabolic pathways of guanine‐containing purines, is linked to cognition phenotype.^38^ A recent study showed that 7‐methylguanine was increased in incident type 2 diabetes mellitus.^39^ Meanwhile, palmitoleate has been suggested to enhance insulin sensitivity, stimulate insulin secretion, increase liver oxidation of fatty acids, improve blood lipid profile, alter the differentiation of macrophages ^40^ and improve metabolic functions in fatty liver tissue through peroxisome proliferator‐activated receptor‐α (PPARα)‐dependent 5′ adenosine monophosphate‐activated protein kinase (AMPK) activation.^41^ Emerging evidence suggests that metabolic impairment is important for AF pathophysiology.^42^ Notably, PPARα‐dependent AMPK activation could result in suppressed inflammation.^43^ Therefore, we speculate that reduction of faecal metabolites such as palmitoleic acid in RAF patients might contribute to excessive inflammation and facilitate AF recurrence. These GM‐related metabolic changes may contribute to the progress of atrial tissue's arrhythmogenic substrate aggravation following catheter ablation. Given the associations of 7‐methylguanine and palmitoleic acid with GM identified in the current work, these two metabolites are speculated to be potential players mediating the impact of GM dysbiosis on RAF progression, at least in part.

Inflammation is associated with multiple pathological events, including oxidative stress, apoptosis and fibrosis, which induce AF substrate generation. Therefore, low‐grade inflammation is considered a potential mechanism contributing to AF. We found the species *Faecalibacterium sp CAG:82* exhibited a decreased trend in the RAF group compared with non‐RAFs. A previous study has demonstrated an anti‐inflammatory effect of gut *Faecalibacterium* through inhibition of interleukin‐6 and transcription 3/interleukin‐17 pathway activation.^44^ Therefore, it is speculated that reduced *Faecalibacterium* abundance in the intestine might increase various inflammatory cytokines, elicit low‐grade inflammation and thus lead to RAF. These interconnected microbial and metabolic changes suggest the involved microbes might contribute to AF recurrence through interactions with specific metabolites in the host.

In addition to the disparity between the non‐RAF and RAF groups, similarities shared by these groups were also revealed in the current study, which may be more important and constitute key events in the onset—but not development—of AF. Furthermore, bacterial organisms and metabolites commonly altered in the non‐RAF and RAF groups were significantly associated. CDCA and sebacic acid were found to be significantly correlated with several taxa. Elevated serum CDCA in the metabolic patterns of non‐RAF and RAF patients has been indicated to have a critical function in the progress of structural remodelling in AF. CDCA is positively correlated with the left atrial low voltage area and promotes apoptosis in atrial myocytes.^45^ Furthermore, sebacic acid belonging to medium‐chain fatty acids is significantly less abundant in both ulcerative colitis and Crohn disease patients.^46^ Therefore, the shared GM and metabolic profile demonstrated above may be associated with or even contribute to AF onset.

These findings provided opportunities to take advantage of the GM for clinical application for improving GM‐related AF pathogenesis,^47^ for example utilizing faecal markers for identifying patients at high risk of RAF. One emerging translational application of the GM is its use as a screening, prognostic or predictive biomarker. Several studies have reported the faecal markers for disease diagnosis, such as colorectal carcinoma, adenoma^47^, ^48^, ^49^ and early‐stage lung cancer.^50^ Apart from their potential for disease detection, associations between bacterial biomarkers and clinical outcome have raised the possibility of using them as markers for treatment prediction and prognostication. For example, the response biomarker to ustekinumab therapy among Crohn's disease patients,^51^ and a GM‐based a set of universal biomarkers for diagnosis, disease activity evaluation and infliximab treatment response prediction in inflammatory bowel disease.^52^ Therefore, the associations between bacterial markers and treatment efficacies or clinical outcome will pave the way to clinically translate the use of GM in the near future. Modulating microorganisms using antibiotics to inhibit disease‐enriched bacteria,^53^ supplementing commensals^54^ or performing faecal microbiota transplantation to replenish disease‐decreased bacteria is also recommended.^55^ These extensive findings will pave the way to translate GM use for clinical intervention, and more studies are imperative to evaluate its clinical value in the context of AF.

In conclusion, the present findings provide a comprehensive description of disordered GM profiles in AF patients with a high risk of recurrence following ablation. More attention might be paid to disordered GM profiles while developing future ablation risk stratification strategies.

## CONFLICTS OF INTEREST

The authors declared no competing interests to this work.

## AUTHOR CONTRIBUTION

**Jing Li:** Conceptualization (equal); Data curation (equal); Funding acquisition (equal); Methodology (equal); Project administration (equal); Resources (equal); Supervision (equal); Writing‐review & editing (lead). **Kun Zuo:** Conceptualization (equal); Data curation (equal); Formal analysis (lead); Investigation (lead); Methodology (equal); Validation (equal); Visualization (lead); Writing‐original draft (lead); Writing‐review & editing (lead). **Jing Zhang:** Investigation (equal); Methodology (equal); Resources (equal). **chaowei hu:** Investigation (equal); Methodology (equal); Resources (equal). **Pan Wang:** Investigation (equal); Methodology (equal); Resources (equal). **Jie Jiao:** Investigation; Methodology; Resources. **Zheng Liu:** Investigation (equal); Methodology (equal); Resources (equal). **Xiandong Yin:** Investigation (equal); Methodology (equal); Resources (equal). **Xiaoqing Liu:** Investigation (equal); Methodology (equal); Resources (equal). **Kuibao Li:** Conceptualization (equal); Data curation (equal); Formal analysis (equal); Methodology (equal); Project administration (equal); Software (equal); Supervision (equal); Visualization (equal); Writing‐review & editing (equal). **Xinchun Yang:** Conceptualization (lead); Funding acquisition (lead); Project administration (lead); Resources (lead); Supervision (lead); Writing‐review & editing (equal).

## Supporting information

Figure S1

Figure S2

Figure S3

Table S1

Table S2

Table S3

Table S4

Table S5

Table S6

Supplementary Material

## Data Availability

The data set holding the results of this study has been uploaded to the European Molecular Biology Laboratory (EMBL) European Nucleotide Archive (ENA) with the BioProject accession code PRJEB28384. All the data described here are available at (https://www.ebi.ac.uk/ena/data/view/PRJEB28384).
