# Supplementary material for: Shifts in gut microbiome and metabolome are associated with risk of recurrent atrial fibrillation
Source: J Cell Mol Med. 2020 Oct 14;24(22):13356–69. doi: 10.1111/jcmm.15959 (PMC7701499; doi:10.1111/jcmm.15959)
Supplement: Supplementary file 1 — Figure S1 [file JCMM-24-13356-s001.pdf]

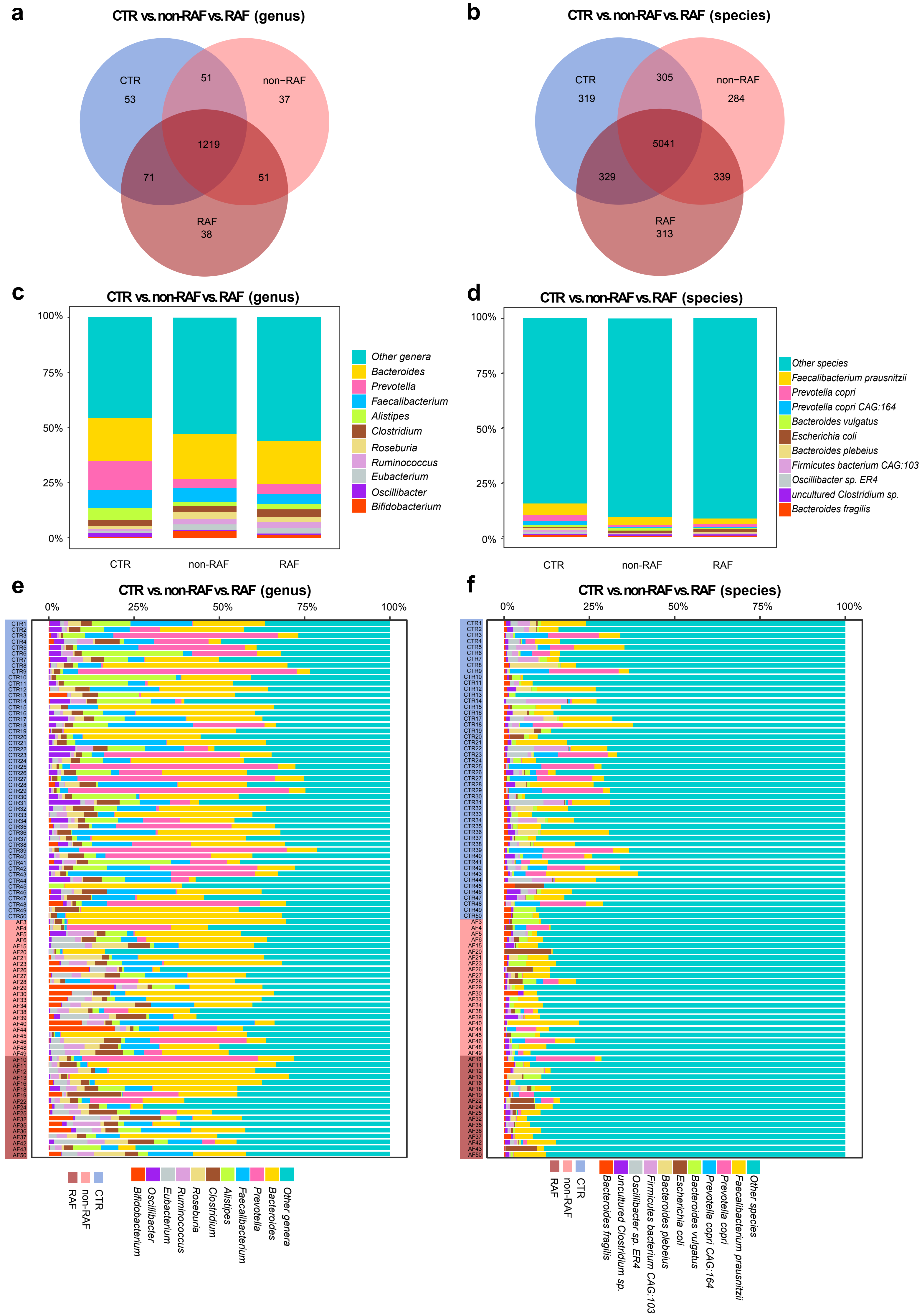
a
CTR vs. non-RAF vs. RAF (genus)
CTR
53
51
non−RAF
37
1219
71
51
RAF
38
b
CTR vs. non-RAF vs. RAF (species)
CTR
319
305
non−RAF
284
5041
329
339
RAF
313
c
CTR vs. non-RAF vs. RAF (genus)
100%
75%
50%
25%
0%
CTR
non-RAF
RAF
Other genera
Bacteroides
Prevotella
Faecalibacterium
Alistipes
Clostridium
Roseburia
Ruminococcus
Eubacterium
Oscillibacter
Bifidobacterium
d
CTR vs. non-RAF vs. RAF (species)
100%
75%
50%
25%
0%
CTR
non-RAF
RAF
Other species
Faecalibacterium prausnitzii
Prevotella copri
Prevotella copri CAG:164
Bacteroides vulgatus
Escherichia coli
Bacteroides plebeius
Firmicutes bacterium CAG:103
Oscillibacter sp. ER4
uncultured Clostridium sp.
Bacteroides fragilis
e
CTR vs. non-RAF vs. RAF (genus)
0%
25%
50%
75%
100%
RAF
non-RAF
CTR
Bifidobacterium
Oscillibacter
Eubacterium
Ruminococcus
Roseburia
Clostridium
Alistipes
Faecalibacterium
Prevotella
Bacteroides
Other genera
f
CTR vs. non-RAF vs. RAF (species)
0%
25%
50%
75%
100%
RAF
non-RAF
CTR
Bacteroides fragilis
uncultured Clostridium sp.
Oscillibacter sp. ER4
Firmicutes bacterium CAG:103
Bacteroides plebeius
Escherichia coli
Bacteroides vulgatus
Prevotella copri CAG:164
Prevotella copri
Faecalibacterium prausnitzii
Other species
