# Supplementary figures and images for: Shifts in gut microbiome and metabolome are associated with risk of recurrent atrial fibrillation
Source: J Cell Mol Med. 2020 Oct 14;24(22):13356–69. doi: 10.1111/jcmm.15959 (PMC7701499; doi:10.1111/jcmm.15959)

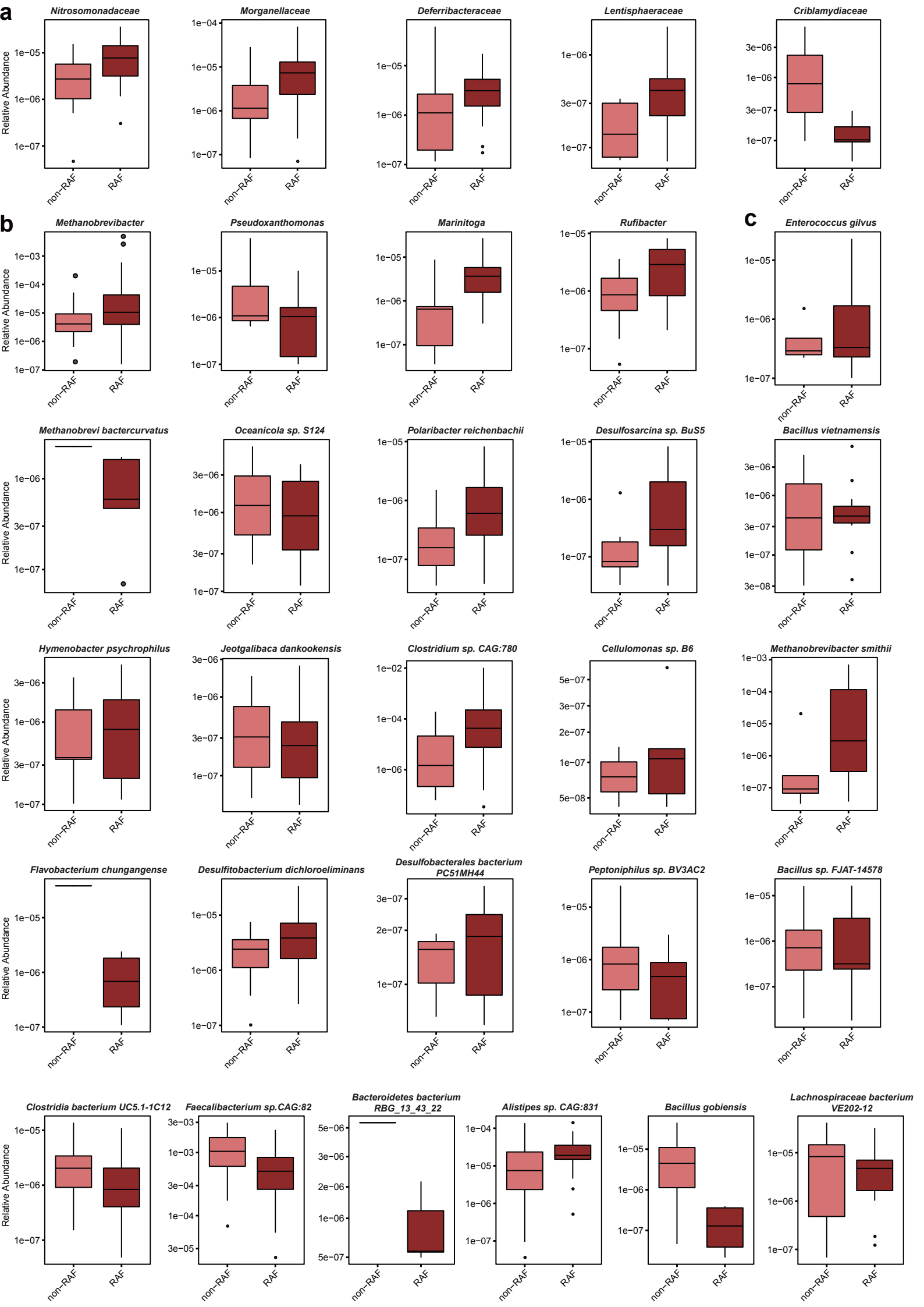

Supplement: Supplementary file 2 — Figure S2 [file JCMM-24-13356-s002.pdf]

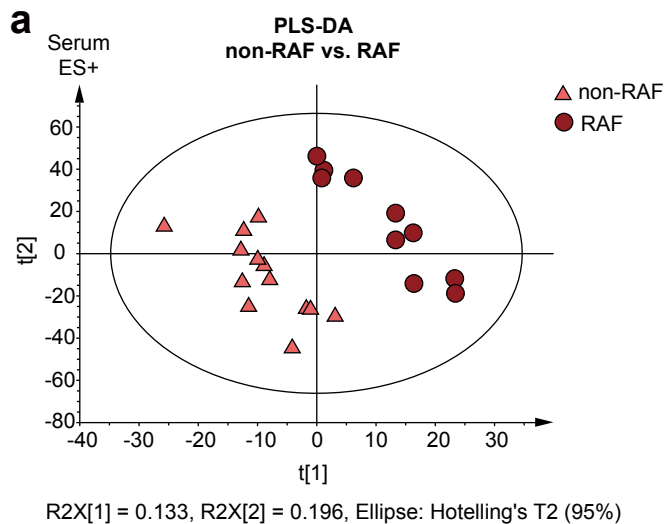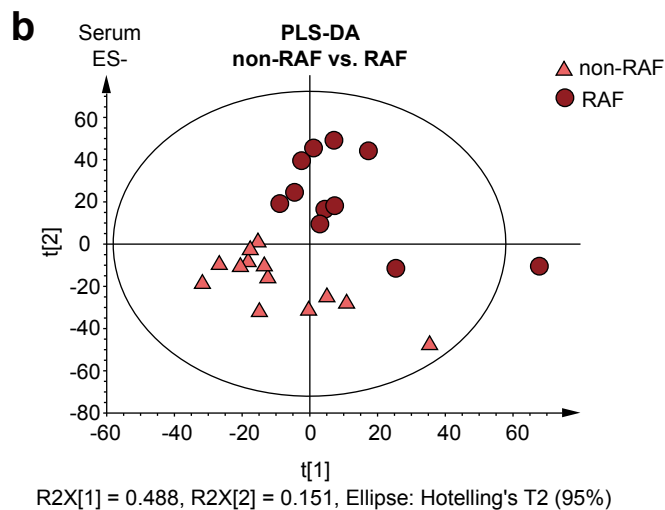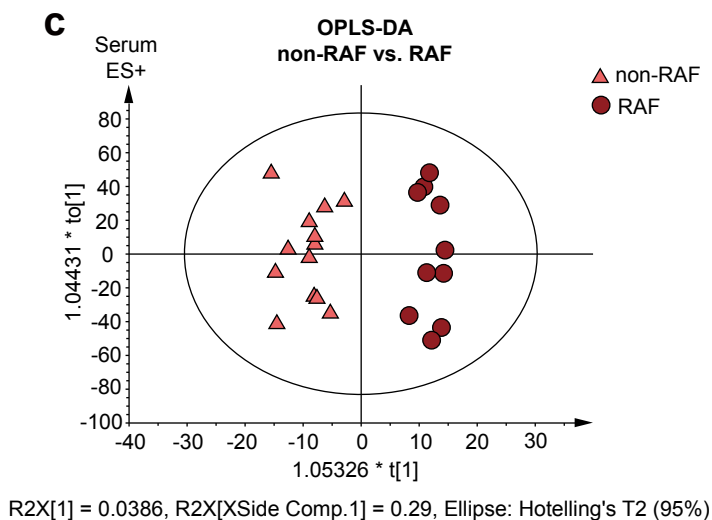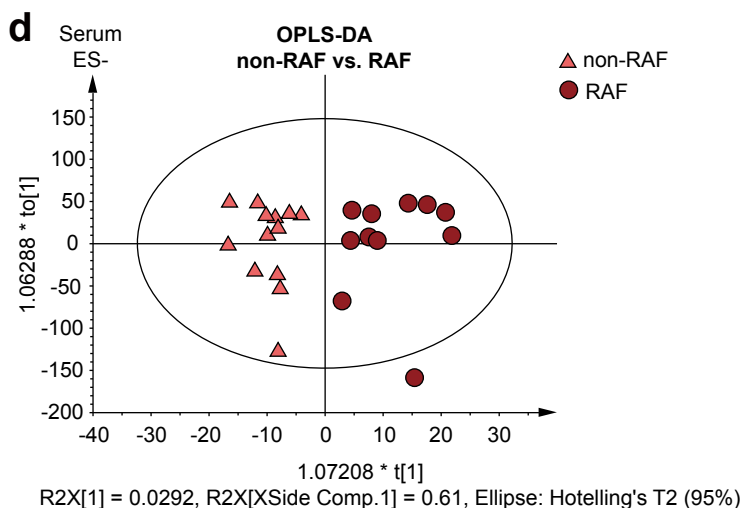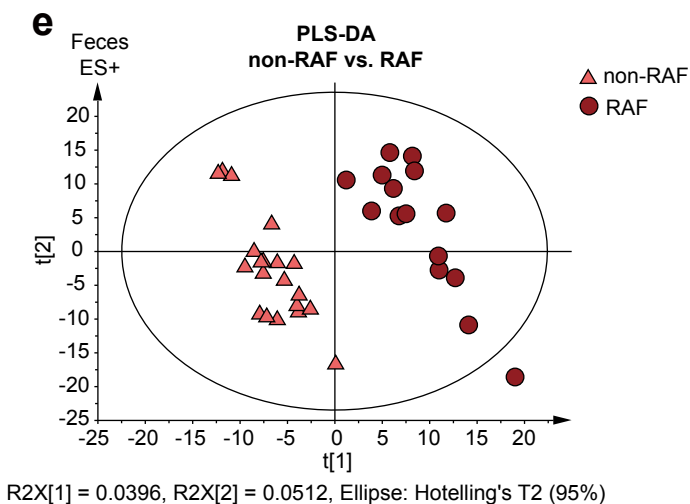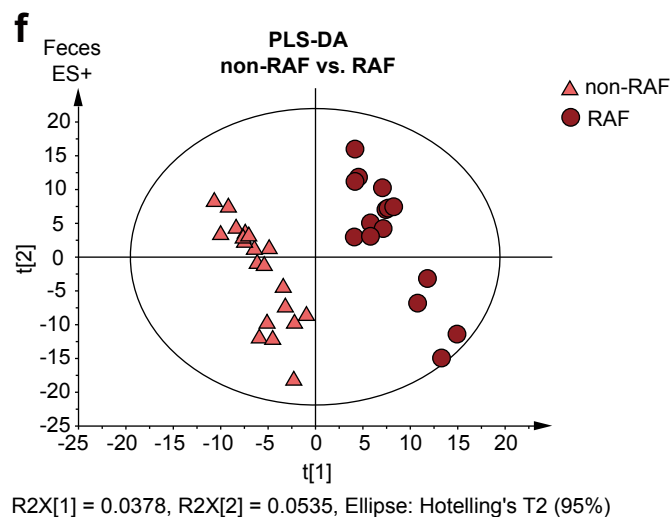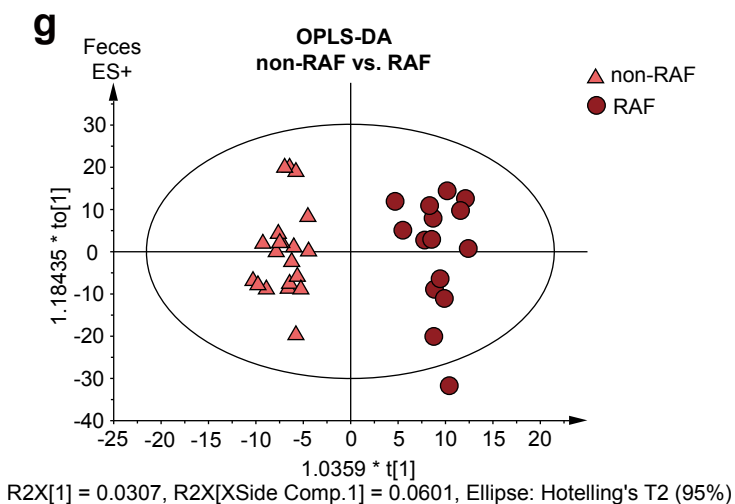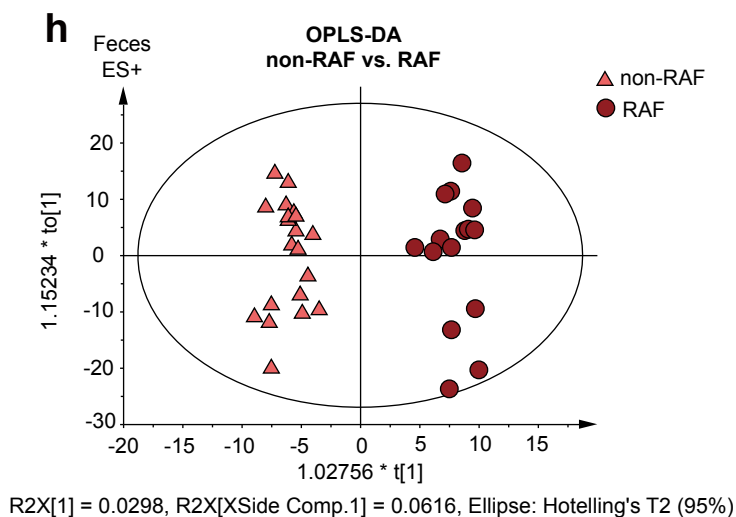

Supplement: Supplementary file 3 — Figure S3 [file JCMM-24-13356-s003.pdf]
