# Supplementary material for: Shifts in gut microbiome and metabolome are associated with risk of recurrent atrial fibrillation
Source: J Cell Mol Med. 2020 Oct 14;24(22):13356–69. doi: 10.1111/jcmm.15959 (PMC7701499; doi:10.1111/jcmm.15959)
Supplement: Supplementary file 9 — Table S6 [file JCMM-24-13356-s009.docx]

**Table S6. Univariate and multivariable Cox regression analysis for predicting AF recurrence.**

| **Factors** | **HR (95% CI)** | **P value** |
| --- | --- | --- |
| **CAAP-AF score** | 1.2 (0.95-1.5) | 0.13 |
| **Tax score** | 2.5 (1.1-5.8) | 0.026 |
| **ACEI** | 1.2 (0.26-5.2) | 0.84 |
| **ARB** | 2.5 (0.68-9.3) | 0.17 |
| **β‐blocker** | 2.8 (0.86-8.9) | 0.086 |
| **CCB** | 0.64 (0.2-2) | 0.45 |
| **statin** | 4.8 (1.3-17) | 0.019 |
| **propafenone** | 3.6 (0.46-28) | 0.23 |
| **amiodarone** | 2.1 (0.74-6.2) | 0.16 |

|  | **Clinical model** | | | | **Combined model** | | | |
| --- | --- | --- | --- | --- | --- | --- | --- | --- |
|  | **P value** | **Hazard ratio** | **Standard error** | **95% CI** | **P value** | **Hazard ratio** | **Standard error** | **95% CI** |
| **CAAP-AF score** | 0.311 | 1.1306 | 0.1371 | 0.8914-1.4339 | 0.590 | 1.066 | 0.1266 | 0.8448-1.3454 |
| **Statin usage** | 0.131 | 2.8897 | 2.0326 | 0.7280-11.4708 | 0.056 | 3.8675 | 2.7373 | 0.9660-15.4844 |
| **Tax score** | / | | | | 0.041 | 2.6473 | 1.2641 | 1.0384-6.7493 |

**Abbreviations:** HR, hazard ratio; CI, confidence interval; CAAP-AF score, CAAP-AF score: coronary artery disease: 1; age: <50: 0, 50-60: 1, 60-70: 2, ≥70: 3; left atrial size: <4: 0, 4-4.5: 1, 4.5-5: 2, 5-5.5: 3, ≥5.5: 4; persistent or longstanding AF: 2; Antiarrhythmics failed: none:0, 1 or 2: 1, >2: 2; and female gender: 1; Tax score: Taxonomic score; ACEI, angiotensin-converting enzyme inhibitors; ARB, angiotensin receptor blockers; CCB, calcium channel blockers.
