# Supplementary material for: Shifts in gut microbiome and metabolome are associated with risk of recurrent atrial fibrillation
Source: J Cell Mol Med. 2020 Oct 14;24(22):13356–69. doi: 10.1111/jcmm.15959 (PMC7701499; doi:10.1111/jcmm.15959)
Supplement: Supplementary file 10 — Supplementary Material [file JCMM-24-13356-s010.docx]

**Supplementary methods:**

For the participants, exclusion criteria were: previous heart failure; coronary atherosclerotic heart disease; structural heart disease; concurrent pathologies such as irritable bowel syndrome, autoimmune disorders, liver disease, kidney diseases or malignancy; antibiotic or probiotic use within a month prior to enrolment. Patient baseline features were collected by face-to-face interviews and from hospital records.

Following catheter ablation, patients underwent systematic follow-up and 12‐lead electrocardiography at 3, 6, 12, and 18 months; respectively; an electrocardiogram would be recorded in case a patient complained of discomfort. Holter monitoring was performed at three- and six- months post-ablation and at six-month intervals afterwards.

In our electrophysiological team, antiarrhythmic medications would be discontinued at five half‐lives or more prior to ablation. After ablation, except for cases with contraindications (e.g., sinus bradycardia, second degree atrial-ventricular block, systolic blood pressure <100 mmHg, hepatic dysfunction and dysthyroidism), all cases with persistent AF and some with paroxysmal AF would receive oral antiarrhythmic drugs for a 3-month period. In the current work, amiodarone and propafenone were administered to 30 (75%) and 7 (17.5%) individuals, respectively.

***GM assessment by Metagenomics***

Genes were predicted from various contigs with MetaGeneMark v12 (GeneMark, USA). Gene abundance was determined by numbering reads after normalization to gene length. Then, DIAMOND v0.7.9.58 was utilized for taxonomic assignments (default settings with the exception of −k 50 −sensitive −e 0.00001). Matches showing statistical significance for various genes with the first hit e≤10 × e-value were utilized for distinguishing taxonomic groups. The taxonomic levels of different genes were assessed with the MEGAN software (MEtaGenomeANalyzer); the abundance levels of different taxonomic groups were evaluated by summing up those of all included genes. Abundance differences were assessed by the Wilcoxon rank sum test, with Benjamini and Hochberg correction. Two-sided P<0.05 indicated statistical significance.

***GM assessment by metabolomics***

Partial least-squares discriminant analysis (PLS-DA) as well as orthogonal partial least-squares discriminant analysis (OPLS-DA) utilized the SIMCA-P software for clustering specimen plots across groups. Compounds with significant between-group changes were determined based on variable effect on projection >1 and P<0.05 according to peak areas.
